# Interpreting T-Cell Cross-reactivity through Structure: Implications for TCR-Based Cancer Immunotherapy

**DOI:** 10.3389/fimmu.2017.01210

**Published:** 2017-10-04

**Authors:** Dinler A. Antunes, Maurício M. Rigo, Martiela V. Freitas, Marcus F. A. Mendes, Marialva Sinigaglia, Gregory Lizée, Lydia E. Kavraki, Liisa K. Selin, Markus Cornberg, Gustavo F. Vieira

**Affiliations:** ^1^Núcleo de Bioinformática do Laboratório de Imunogenética (NBLI), Department of Genetics, Universidade Federal do Rio Grande do Sul (UFRGS), Porto Alegre, Brazil; ^2^Kavraki Lab, Department of Computer Science, Rice University, Houston, TX, United States; ^3^Laboratório de Imunologia Celular e Molecular, Instituto de Pesquisas Biomédicas (IPB), Pontifícia Universidade Católica do Rio Grande do Sul (PUCRS), Porto Alegre, Brazil; ^4^Lizée Lab, Department of Melanoma Medical Oncology – Research, The University of Texas M. D. Anderson Cancer Center, Houston, TX, United States; ^5^Selin Lab, Department of Pathology, University of Massachusetts Medical School, Worcester, MA, United States; ^6^Cornberg Lab, Department of Gastroenterology, Hepatology and Endocrinology, Hannover Medical School, Hannover, Germany; ^7^German Center for Infection Research (DZIF), Partner-Site Hannover-Braunschweig, Hannover, Germany; ^8^Programa de Pós-Graduação em Saúde e Desenvolvimento Humano, Universidade La Salle, Porto Alegre, Brazil

**Keywords:** T-cell cross-reactivity, peptide–MHC complex, cross-reactivity hot-spots, TCR-interacting surface, hierarchical clustering, TCR/pMHC, cancer immunotherapy

## Abstract

Immunotherapy has become one of the most promising avenues for cancer treatment, making use of the patient’s own immune system to eliminate cancer cells. Clinical trials with T-cell-based immunotherapies have shown dramatic tumor regressions, being effective in multiple cancer types and for many different patients. Unfortunately, this progress was tempered by reports of serious (even fatal) side effects. Such therapies rely on the use of cytotoxic T-cell lymphocytes, an essential part of the adaptive immune system. Cytotoxic T-cells are regularly involved in surveillance and are capable of both eliminating diseased cells and generating protective immunological memory. The specificity of a given T-cell is determined through the structural interaction between the T-cell receptor (TCR) and a peptide-loaded major histocompatibility complex (MHC); i.e., an intracellular peptide–ligand displayed at the cell surface by an MHC molecule. However, a given TCR can recognize different peptide–MHC (pMHC) complexes, which can sometimes trigger an unwanted response that is referred to as T-cell cross-reactivity. This has become a major safety issue in TCR-based immunotherapies, following reports of melanoma-specific T-cells causing cytotoxic damage to healthy tissues (e.g., heart and nervous system). T-cell cross-reactivity has been extensively studied in the context of viral immunology and tissue transplantation. Growing evidence suggests that it is largely driven by structural similarities of seemingly unrelated pMHC complexes. Here, we review recent reports about the existence of pMHC “hot-spots” for cross-reactivity and propose the existence of a TCR interaction profile (i.e., a refinement of a more general TCR footprint in which some amino acid residues are more important than others in triggering T-cell cross-reactivity). We also make use of available structural data and pMHC models to interpret previously reported cross-reactivity patterns among virus-derived peptides. Our study provides further evidence that structural analyses of pMHC complexes can be used to assess the intrinsic likelihood of cross-reactivity among peptide-targets. Furthermore, we hypothesize that some apparent inconsistencies in reported cross-reactivities, such as a preferential directionality, might also be driven by particular structural features of the targeted pMHC complex. Finally, we explain why TCR-based immunotherapy provides a special context in which meaningful T-cell cross-reactivity predictions can be made.

## 1 Hypothesis and Theory

### 1.1 Cellular Immunity, Private Specificity, and T-Cell Cross-reactivity

Cellular immunity relies on T-cell lymphocytes and their ability to produce unique T-cell receptors (TCRs), while humoral immunity relies on B-cell lymphocytes and their ability to produce antibodies (also referred to as B-cell receptors) (1, 2). Combined, these two branches compose the adaptive immunity, a major “upgrade” in the evolution of the immune system, first seen in jawed vertebrates (1, 2). Different from more ancestral mechanisms of innate immunity, adaptive immunity allows creating specific immune responses to virtually any new pathogen encountered by the host organism. It also allows generating immunological memory, protecting the host against future encounters with the same pathogen (3). This new system was essential in facing the threat of viruses, which are incredibly diverse and evolve at an amazing rate (4). While antibodies can neutralize circulating viruses, cytotoxic T-cells can find and eliminate infected cells (i.e., the “hijacked factories” producing new viral particles). In fact, coevolution with viruses is a major factor shaping the complexity and diversity of the mechanisms involved in cellular immunity (5–7).

The key players in this system are the major histocompatibility complex (MHC) molecules, a diverse set of protein receptors capable of binding peptides derived from intracellular proteins and displaying them at the cell surface (5). This allows circulating cytotoxic T-cells to interact directly with these peptide–MHC (pMHC) complexes, using their TCRs. After a complex selection process in early stages of their development (8, 9), T-cells are able to recognize “non-self” pMHC complexes. For instance, a virus-infected cell displays at its surface MHC molecules loaded with virus-derived peptides. These non-self pMHC complexes can trigger a T-cell response that, in turn, eliminates the infected cell. Moreover, the recognition of these non-self pMHCs can generate immunological memory against this particular virus strain (3).

The efficiency of antiviral immunity, however, depends on the ability of an individual to produce and store a pool of memory T-cells (i.e., a T-cell *repertoire*) able to specifically recognize most of the hugely variable pMHC complexes displayed by cells in different tissues. It actually is quite a puzzling task, if one considers (i) the diversity of MHC allotypes of the host (i.e., the number of MHC protein variants in the human population), (ii) the genetic variability of viruses (i.e., peptide diversity), and (iii) the frequency of viral infections. The solution to this puzzle involves a combination of two important features of cellular immunity: (i) *somatic recombination* of TCR-encoding genes and (ii) *T-cell cross-reactivity*. Somatic recombination allows for a potential combinatorial diversity of TCRs which exceeds 10^20^ (10, 11). Cross-reactivity allows optimizing the repertoire of T-cells for the recognition of most possible targets, despite the limited number of T-cells that can exist in a given individual, at a given time (≈10^11^ in humans) (10, 12). Each newly generated T-cell has a unique TCR and is added to the diverse repertoire of circulating T-cells. If activated by a given pMHC, one T-cell generates an entire pool of clone cells (referred to as a *T-cell line*). All these clones display essentially the same TCR and, therefore, are specific to the same (*cognate*) pMHC. However, after being added to the memory pool, some of these T-cells can be recruited in an initial response to a different *heterologous* pMHC (e.g., the same MHC displaying the peptide of a different virus).

T-cell cross-reactivity is defined as the ability of a given T-cell to be activated by two or more heterologous pMHCs (12). This cross-reactivity can even mediate *heterologous immunity*, when a contact with one pathogen generates a partial immunity against a second (heterologous) pathogen (13). Heterologous immunity is a double-edged sword: it can be protective and desired for wide spectrum vaccine development (14, 15), but it can also mediate impaired cellular response, chronic infection and immunopathology (15–18). The stochastic nature of TCR specificity generation entails that each individual has a unique set of TCRs (referred to as *private specificity*) (13). In addition, given the size limit of the T-cell repertoire and the constant challenges with a variety of pathogens, the memory pool of an individual is ever changing (e.g., some T-cell lines expand, others are lost) (19, 20). In time, cross-reactive cells represent an important part of our memory repertoire, and our immunity against every new challenge is directly influenced by our *immunological history* (12, 19, 21–23). Note that there exist some known biases in the somatic recombination process, producing some TCR sequence combinations with higher frequency in a population (24). This phenomenon is referred to as *public TCR usage* and will be discussed later (see section 1.4).

Recent studies are corroborating the idea that T-cell cross-reactivity is the rule, rather than the exception (19, 22, 25, 26), and that structural features involved in specific TCR/pMHC interactions are the main features driving cross-reactive responses against heterologous targets (25, 27–29). Despite all the evidence accumulated in the context of viral immunity and tissue transplantation, integration of T-cell cross-reactivity into other fields of immunology and human health has been rather slow. This delay can be partially explained by the complexity of the mechanisms involved, as well as concerns about the reproducibility of experimental results characterizing T-cell cross-reactivity (26).

In a pioneering study, Wedemeyer and colleagues were able to collect T-cells recognizing a peptide derived from hepatitis C virus (HCV), from the blood of healthy donors (30) who had no history of infection by HCV. This implied that these HCV-specific T-cells were probably cross-reactive memory cells previously triggered by a heterologous pathogen. In fact, the authors were able to identify a peptide from influenza A virus (IAV) having 77% of sequence similarity with the HCV-derived peptide used to expand the T-cells. They also showed that these cells were able to recognize both peptides, and that T-cells with the same specificity were generated in response to IAV infection. However, a later study by Kasprowicz et al. (31) suggested that cross-reactivity between these heterologous peptides was rather weak and had a preferential *directionality* from HCV to IAV (i.e., T-cells primed with the HCV-derived peptide also recognize the IAV-derived peptide, but the opposite was usually not true) (31). More recent studies help clarify situations like this, showing that heterologous immunity between viruses is greatly influenced by private specificities and immunological history (19, 23, 32). Therefore, observed results are not solely determined by peptide sequence similarity, but also dependent on the particular T-cells dominating the response (*in vivo*), or the T-cell line selected for the experiments (*in vitro* or *ex vivo*) (24).

The rebirth of T-cell cross-reactivity as a major interest for human health, however, is coming from cancer research. For decades, immunologists have suggested that the same mechanisms involved in antiviral surveillance were also involved in detecting and eliminating cancer cells, which can display MHCs loaded with tumor-specific peptides (33). More recently, the field of cancer immunotherapy has grown as one of the most promising paths for cancer treatment, relying on the mechanisms of cellular immunity to provide personalized therapies that can eliminate tumors in different tissues and even generate protective memory (33–36). A number of TCR-based therapies were put forward, making use of the latest molecular biology technologies to enhance TCR affinity against tumor-specific peptides (37). Unfortunately, the excitement was tempered by safety concerns. These supposedly tumor-specific T-cells can present unexpected T-cell cross-reactivities in some individuals, attacking healthy tissues (38). In fact, off-target toxicity effects have been observed in recent clinical trials, with at least 5 deadly cases reported (39–41). Two of these cases were clearly linked to T-cell cross-reactivity between the targeted tumor-specific peptide (the melanoma-associated antigen MAGE-A3) and a Titin-derived peptide expressed in healthy cardiac cells (39, 42). The peptides involved have only 55% of sequence similarity, exemplifying the great challenge faced by current preclinical screenings. Later analysis using X-ray crystallography confirmed the structural similarity of the corresponding pMHC complexes as the molecular basis for the observed T-cell cross-reactivity (43).

In response to this critical need, new computational approaches are being developed and tested to improve our capacity to screen for potentially dangerous cross-reactivities. Some of these methods involve assessing peptide sequence similarity, while also accounting for protein tissue expression and MHC binding (44, 45). Others are based on pMHC structural similarity (46–48) or some combination of previously mentioned features (49, 50). Despite the incredible challenge at hand and the current limitations of these computational methods, encouraging results are being reported. For instance, some of these methods can predict the previously mentioned cross-reactivity between the peptides derived from MAGE-A3 and Titin. A better understanding of the mechanisms underlying T-cell cross-reactivity, as well as the relationship between structural features of pMHC complexes and the activation of T-cell clones, is of upmost importance to further improve these computational methods. In turn, such progress will allow us to provide useful predictions that can be directly translated to the clinic.

In the following sections we attempt to connect the dots between the current understanding of pMHC structure and the goal of making safer TCR-based immunotherapies. First, we review structural aspects of the TCR/pMHC interaction and introduce the idea of structural clustering of pMHC complexes (section 1.2). Then we apply clustering methods to both available crystallographic data and modeled pMHC complexes, providing further evidence that pMHC structural information is essential to understand T-cell cross-reactivity (section 1.3). Next, we review how structural features of the pMHC complex can actually shape the TCR repertoire (section 1.4). Going one step further, we hypothesize how the same features might be shaping different patterns of cross-reactivity: they can be responsible for weak cross-reactivity among similar peptide-targets (section 1.5), or, conversely, drive cross-reactive responses among completely unrelated peptide-targets (section 1.6). Finally, we consider the implications of our work for T-cell cross-reactivity prediction and discuss why cancer immunotherapy provides a special context in which meaningful progress can be made (section 1.7).

### 1.2 Structural Analyses Can Uncover Key Features for T-Cell Activation

For simplicity, we usually talk about cross-reactivity of TCRs that recognize different peptides, but it is important to keep in mind that the TCR does not recognize the peptide itself; it recognizes the combined surface of the pMHC complex (51). Therefore, observed cross-reactivities between peptides are linked to their presentation in the “context” of a particular MHC. Even if two different MHCs are capable of binding the same peptide, which is not common, the resulting pMHC complexes will most likely be different (52). In fact, this is one of the causes for rejection in (allogeneic) tissue transplantation (26, 53). In this study, we focus on cross-reactivity between peptides presented by the same class I MHC. However, cross-reactivity involving different MHCs has also been reported (53, 54), and the discussion presented here can also be extended to that context.

Studies using X-ray crystallography have greatly contributed to the current understanding of the TCR/pMHC interaction, which was recently reviewed by Degauque et al. (26). The TCR structure contains flexible loops that can come in contact with the *TCR-interacting surface* of the pMHC (i.e., the “face” of the pMHC complex exposed to TCR interaction; see Figures S1A–C in Supplementary Material). These loops include the complementarity-determining regions (CDRs), which are the most variable regions of the TCR structure and the result of the previously mentioned somatic recombination. Despite the structural flexibility of these loops and the possibility of local conformational changes (25, 55), there is a conserved binding mode for the TCR/pMHC complex. Most times, the CDRs corresponding to the *α* chain of the TCR will interact with the amino-terminal portion of the peptide, while the *β* chain CDRs will interact with the carboxi-terminal of the peptide, at a particular angle (26, 51) (Figure S1D in Supplementary Material). Note that the general docking mode of a TCR to its cognate pMHC is referred to as the *TCR footprint* (51). Although the mechanisms are still open for debate, recent studies suggest that the orientation of the TCR footprint is guided by genetically imprinted biases (on the TCR) to recognize conserved MHC amino acid residues (i.e., *germline bias*) (26, 29). However, with the accumulation of crystal structures and evidence from new experimental approaches, one can also see that different TCRs establish different interaction networks, and that some interactions on the pMHC surface seem more important than others to trigger recognition by a particular T-cell (24, 29). These special contacts have been previously referred to as *hot-spots* for T-cell cross-reactivity (25, 29, 56).

In previous work, our group described an *in silico* approach to evaluate the structural similarity of pMHC complexes (46, 48). We used hierarchical clustering as a tool to group pMHC complexes according to the similarity of their TCR-interacting surfaces. We also used available crystal structures as a reference to implement a method to model pMHC complexes for which no structural data were available (52, 57). Combining these methods, we were able to reproduce experimentally observed cross-reactivity patterns for a dataset of 28 naturally occurring variants of an HCV-derived peptide used for vaccine development (CINGVCWTV) (46). We also applied these methods to predict potential cross-reactivities between this HCV vaccine peptide and a dataset of non-related virus-derived peptides, in the context of a particular human MHC (HLA-A*02:01) (46). Our predictions were later confirmed by *in vitro* and *ex vivo* experiments (47), highlighting the prospecting potential of our methods. One of the detected cross-reactive peptides, derived from Epstein–Barr virus (LLWTLVVLL), shared no sequence similarity with the vaccine peptide. Notwithstanding, both peptides show remarkably similar TCR-interacting surfaces when bound to HLA-A*02:01 (46, 47).

### 1.3 Structural Similarity of pMHC Complexes Can Reveal Their Likelihood for T-Cell Cross-reactivity

**Figure 1:**
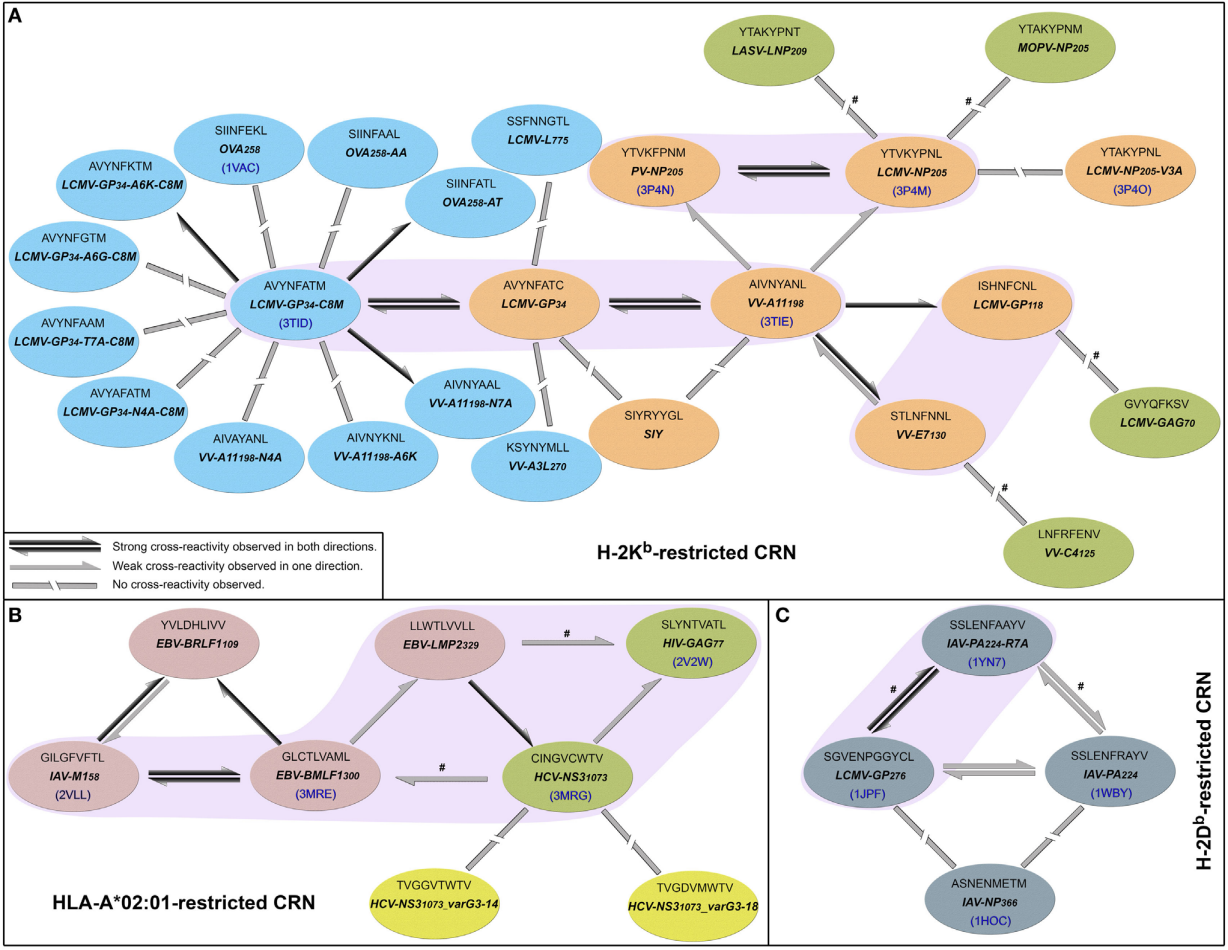
Cross-reactivity networks (CRNs) compiled from previous publications. Arrows indicate the directionality of reactions observed experimentally, with colors indicating stronger (black) or weaker (gray) responses. Segmented connectors indicate non-cross-reactive targets. Each ellipse represents one peptide in the context of **(A)** murine H-2K*^b^*, **(B)** human HLA-A*02:01, or **(C)** murine H-2D*^b^* MHC allotypes. Each ellipse contains the peptide sequence, abbreviation, and PDB code (when available). Ellipses’ colors indicate the source of the cross-reactivity information. Most data were compiled from Cornberg et al. (22) (orange and red ellipses) and expanded with data from Shen et al. (28) (cyan) and Fytili et al. (60) (yellow). Gray ellipses indicate data from Wlodarczyk et al. (17), and dark green ellipses indicate targets included based on sequential/structural analyses (see Methods and Resources). The symbol # was used to indicate reactions suggested by sequential/structural analyses that were not yet tested *in vitro*/*in vivo*. Purple areas indicate complexes with greater structural similarity according to our hierarchical clustering analyses.

In 2010, Cornberg et al. (22) described *cross-reactivity networks* involving virus-derived peptides, within both human and murine memory T-cell pools (CD8^+^/CD44*^hi^*). They used as a reference a peptide derived from vaccinia virus (VV), corresponding to a 9-mer sequence starting at position 198 of the A11 protein (hereafter denoted by VV-A11_198_). Using this VV-derived peptide, which is displayed by the murine MHC H-2K*^b^*, the authors were able to activate three different memory T-cell populations that also recognized peptides from lymphocytic choriomeningitis virus (LCMV-GP_34_, LCMV-GP_118_, and LCMV-NP_205_). Therefore, VV-A11_198_ could be seen as a cross-reactivity “hub,” connected to all these LCMV-derived peptides (Figure 1A). The concept of cross-reactivity networks is interesting in highlighting how broad these T-cell cross-reactivities can be (25), sometimes involving completely unrelated targets. In this sense, graphical representations of such networks have been used in previous works (13, 50, 58, 59). However, it is extremely important to keep in mind that despite providing a nice way to visually summarize cross-reactivity relationships, the topology of these networks might not correspond to the cross-reactivities observed for a particular T-cell line. In other words, the “real” topology of the network in terms of T-cell activation depends on which T-cell is used to test these peptide-targets. In this study, we use cross-reactivity networks to summarize the information from previous studies, as a reference to analyze structural data and discuss cross-reactivity patterns (Figure 1). In our representation, each node describes a given peptide, and only peptides displayed by the same MHC are included in a given network (i.e., MHC-restricted network). Note that this is a schematic representation of the known relationships among peptides that are relevant to our discussion, and not a complete picture of known cross-reactivities; it is not expected to reflect the patterns observed in any particular T-cell assay. Additional information on all peptides included in our analysis can be found in Table S1 in Supplementary Material.

**Figure 2:**
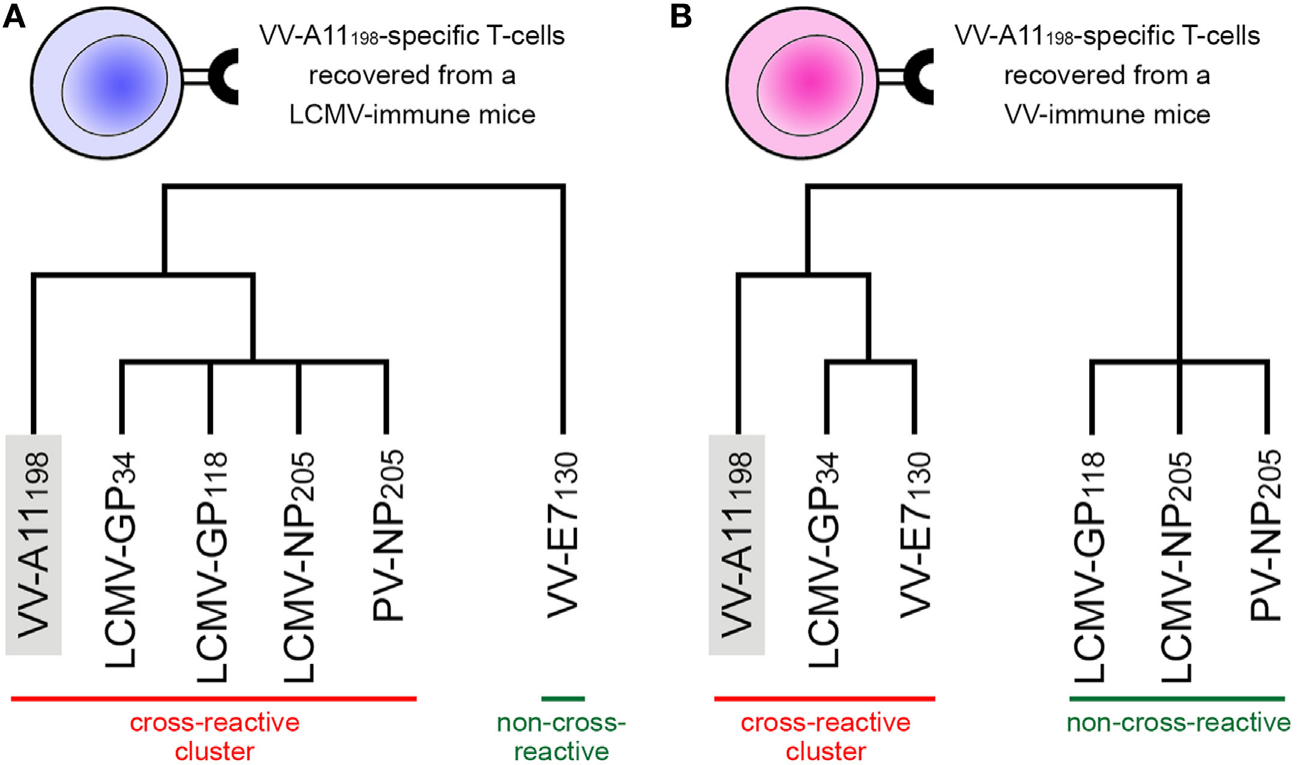
Schematic representation of experimentally observed cross-reactivity patterns. Two alternative dendrograms were drawn to represent alternative outcomes observed in experiments previously performed by Cornberg et al. (22). **(A)** VV-A11_198_-specific T-cells recovered from mice previously immunized with lymphocytic choriomeningitis virus (LCMV) recognize the cognate peptide (indicated by the gray box) as well as three other peptides derived from LCMV and one derived from pichinde virus (PV). We can represent these connections as a “cross-reactivity-cluster” in our dendrogram, as indicated in red. Another peptide derived from vaccinia virus (VV-E7_130_), however, is not recognized. **(B)** VV-A11_198_-specific T-cells recovered from mice previously immunized with vaccinia virus (VV) recognize the cognate peptide (gray box) as well as the other VV-derived peptide (VV-E7_130_) and one LCMV-derived peptide (LCMV-GP_34_). However, in this experiment, no cross-reactivity was observed against peptides LCMV-GP_118_, LCMV-NP_205_, and PV-NP_205_ (indicated by the green bar). Although targeting the same VV-derived peptide, the alternative cross-reactivity patterns described in panels **(A,B)** reflect the use of different T-cell lines in each experiment (indicated as a blue or pink T-cell). Note that cross-reactivity between VV-A11_198_ and LCMV-GP_34_ was observed in both experiments, suggesting higher structural similarity of these peptides when displayed by H-2K*^b^*. All peptides involved in these experiments are restricted to the murine MHC H-2K*^b^*. This is a schematic representation, and the heights of the edges in the dendrogram do not capture the actual “distances” among the peptide-targets. Additional information on the presented peptides can be found in Table S1 in Supplementary Material.

In their original study, Cornberg et al. (22) suggested that observed cross-reactivity patterns present a within-individual variation driven by private specificities and immunological history. For instance, the authors were able to collect VV-A11_198_-specific T-cells from mice previously immunized with LCMV (i.e., LCMV-immune mice). Note that if the donor had no previous contact with VV-derived peptides, these VV-A11_198_-specific T-cells should be cross-reactive cells primarily expanded *in vivo* by recognizing some LCMV-derived target. These cells were further expanded *in vitro* with the cognate (VV-A11_198_) peptide and challenged with different peptides derived from LCMV, VV and pichinde virus (PV). Interestingly, these VV-A11_198_-specific T-cells presented cross-reactivity with LCMV-GP_34_, LCMV-GP_118_, LCMV-NP_205_, and PV-NP_205_ (22) (Figure 2A). However, cross-reactivity against another VV-derived peptide (VV-E7_130_) was not observed. On the other hand, a very different pattern was observed when the authors performed a similar experiment, but expanding VV-A11_198_-specific T-cells from VV-immune mice instead of LCMV-immune mice (Figure 2B). In this case, cross-reactivity with VV-E7_130_ and LCMV-GP_34_ was observed, but no cross-reactivity was observed with LCMV-GP_118_, LCMV-NP_205_, and PV-NP_205_. These contrasting results suggest the use of a different T-cell population with a different specificity (22). They also suggest a greater structural similarity between VV-A11_198_ and LCMV-GP_34_, since this cross-reactivity was observed for both LCMV-immune and VV-immune background. In fact, structural similarity between these targets was later confirmed by Shen et al. (28), which solved the crystal structures of VV-A11_198_ and LCMV-GP_34_-C8M bound to H-2K*^b^* (PDB codes 3TIE and 3TID, respectively).

Out of the 25 pMHCs included in our H-2K*^b^*-restricted network (Figure 1A), at the time of our analysis, only 6 had their structure determined by experimental methods. Using our previously described structure-based approach (46), we performed a hierarchical clustering of these 6 crystallographic structures (Figure S2 in Supplementary Material). Supported by multiscale bootstrap resampling with the R package *pvclust* (61), the clustering agreed with experimental data. The cross-reactive targets VV-A11_198_ and LCMV-GP_34_ fall in the same cluster; the same is observed for the highly cross-reactive targets LCMV-NP_205_ and PV-NP_205_. These four targets are closer to one another than to the non-cross-reactive target OVA_258_. Finally, the most different structure in this analysis contained the non-cross-reactive escape variant LCMV-NP_205_-V3A (21, 62).

**Figure 3:**
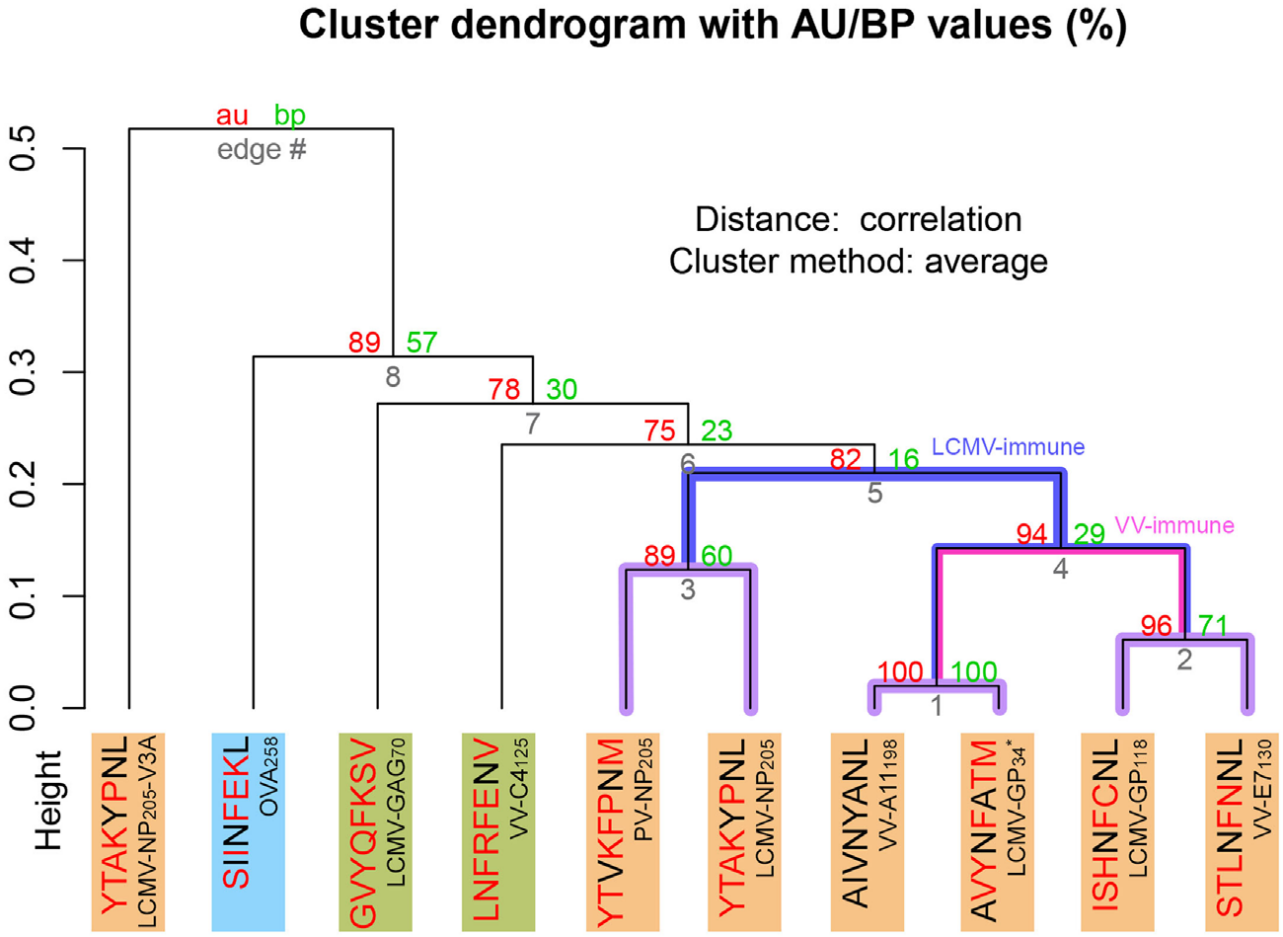
Extended H-2K*^b^*-restricted clustering. Structure-based hierarchical clustering performed with pvclust (61). Each putative cluster is represented by a specific edge (gray numbers), in order of increasing heights (y axis). Cluster confidence is measured with two p-values, approximately unbiased (AU), and bootstrap probabilities (BP). Lines highlighted in purple indicate structures with greater structural similarity (as represented in Figure 1). Lines highlighted in blue and pink indicate putative cross-reactivity thresholds for different memory T-cells (see Figure 2). Each peptide target is colored according to Figure 1. Peptide abbreviation and sequence are provided, with red amino acids indicating changes in relation to VV-A11_198_. *Crystal structure 3TID was used to represent LCMV-GP_34_, despite presenting a C8M exchange, as indicated by its sequence (see Methods and Resources).

To expand our analysis, we used the pMHC modeling method implemented in DockTope (52, 57), obtaining the structures of other complexes previously tested by Cornberg et al. (22) (Figure 1A). We also included in this analysis two unrelated peptides, VV-C4_125_ and LCMV-GAG_70_, as putative non-cross-reactive controls (Table S1 in Supplementary Material). Our expanded hierarchical clustering reflects the greater structural similarity between VV-A11_198_ and LCMV-GP_34_, since both complexes fall in the same cluster, with the edge presenting the lowest height and the highest *p*-values (Figure 3). Peptides LCMV-GP_118_ and VV-E7_130_, which are cross-reactive with VV-A11_198_, fall in the next branch, followed by a cluster with the other cross-reactive targets (LCMV-NP_205_ and PV-NP_205_). All these cross-reactive targets were grouped into a bigger cluster (see edge 5 in Figure 3), apart from all the non-cross-reactive targets. As discussed by Cornberg et al. (22), these cross-reactivities could not be easily predicted with peptide sequence similarity, since all these peptides share less than 50% of their amino acid residues. For instance, sequence similarity between VV-A11_198_ and LCMV-GP_34_ is only 37.5%, the same as between VV-A11_198_ and the non-cross-reactive target OVA_258_. In spite of that, our results show that this cluster of cross-reactivity involving peptides from three different viruses could be predicted by an *in silico* analysis of the corresponding pMHC structures (see edge 5 in Figure 3).

Cross-reactivity was indeed observed among these 6 peptides in the context of H-2K*^b^* (22, 28). These pMHC complexes also present structural similarities, being clustered together in our structure-based hierarchical clustering. However, there was no experimental evidence of one T-cell population able to recognize all six peptides (22). As already discussed, cross-reactivity patterns depend on the specific T-cell population tested. Assuming our clustering correctly captures the relationships among these pMHCs, in terms of structural similarity, we can make some inferences about the T-cells used in the aforementioned experiments. We can say that T-cells from LCMV-immune mice are more cross-reactive, and we can visually represent them with a higher threshold in our clustering analysis (defining the blue cluster in Figure 3). Such threshold would correctly predict most of the observed cross-reactivities, with the exception of VV-E7_130_ (which was not recognized). On the other hand, T-cells from VV-immune mice can be represented with a lower threshold (defining the pink cluster in Figure 3), since VV-A11_198_-specific T-cells recognize neither NP_205_ peptides. The exception in this case, would be LCMV-GP_118_. These exceptions cannot be predicted considering the information provided by the pMHC structures, since they are most likely driven by TCR variability and private specificities. In spite of that, our data suggest a correlation between pMHC structural similarity and the probability to find cross-reactivity among pMHC targets; that is, the higher the similarity, the higher the likelihood of observing cross-reactive responses. Although cross-reactivity between LCMV-GP_118_ and VV-E7_130_ was not observed using the VV-A11_198_-specific or VV-E7_130_-specific T-cells (22), the similarity of these pMHC complexes (Figure S3 in Supplementary Material) suggests that this cross-reactivity should be observed using another T-cell population; maybe with LCMV-GP_118_-specific T-cells.

### 1.4 Structural Features of the pMHC Can Shape the TCR Repertoire

More than a decade ago, Turner and colleagues (63) described differences in the T-cell population stimulated by a featureless peptide (referred to as a “vanilla” peptide), and a peptide having a prominent feature exposed to the TCR (hereafter referred to as a “spicy” peptide). The authors used a peptide derived from the polymerase acidic protein of influenza A virus as an example of spicy peptide (IAV-PA_224_, see Table S1 in Supplementary Material). This peptide has an arginine at position 7 (P7), which becomes an exposed feature when displayed by the murine MHC molecule H-2D*^b^* (Figure S4 in Supplementary Material). Immunization with this peptide triggered the expansion of a very diverse pool of T-cells, including cells with high affinity to the target pMHC. Comparing the response across different animals, the authors noticed great variability in TCR usage. In other words, in each animal the response was dominated by TCRs with unique CDR sequences (i.e., shaped by private specificity).

Surprisingly, opposite results were observed when using a vanilla peptide. Immunization with a mutated version of IAV-PA_224_, replacing the arginine at P7 with an alanine (IAV-PA_224_-R7A), triggered the expansion of a much less diverse T-cell population. In this case, similar CDR sequences were observed for different individuals (i.e., public TCR usage). The same results were observed with a wild-type vanilla peptide (IAV-NP_366_). Therefore, structural features of the pMHC complex can shape the composition of the TCR repertoire during a cellular immune response. A pMHC displaying a vanilla peptide has a TCR-interacting surface dominated by the (self) MHC; given the negative selection of T-cells, very few available TCRs can recognize this complex. This could explain the observation of a less diverse population and the use of public TCRs, sharing a germline bias to interact with the MHC. In addition, we could expect such TCRs to be more cross-reactive, since they rely mostly on (self) MHC features for the recognition. On the other hand, a spicy peptide offers a more evident discerning feature that various TCRs can recognize (in slightly different ways). Given their “focus” on this outstanding feature, we could expect such TCRs to be intrinsically less cross-reactive and they should be incapable (or impaired) to recognize pMHCs lacking such feature.

It is easier to understand this analogy of the spicy feature having in mind some prominent structure that is specific to the peptide, as the examples mentioned earlier and in the next section. However, the TCR/pMHC interaction can be influenced by more subtle features, as recently described by Song et al. (24). They performed a comprehensive evaluation of the T-cell response to the peptide IAV-M1_58_, displayed by HLA-A*02:01, using the next-generation sequencing of TCRs. In addition, they resolved the crystal structures of two selected TCR/pMHC complexes. IAV-M1_58_ has been described as a vanilla peptide, since most of its side chains are buried when displayed by HLA-A*02:01. In turn, it was suggested that the lack of recognizable peptide features would lead to a very narrow T-cell response (i.e., lack of TCR diversity among stimulated T-cells). However, Song et al. (24) observed that the IAV-M1_58_:HLA-A*0201 complex can actually be recognized by a broad range of TCRs; most of them sharing the same V*β* domain. They were also able to identify a conserved structural feature that seemed to be required for the recognition of this peptide. Interestingly, it was not something “prominent,” and it was not exactly a feature of the peptide alone. In fact, the authors describe a unique exposed pocket between the peptide and the MHC, with which very different TCRs are able to interact. In other words, this particular pocket is a recognizable structural feature that is specific to the IAV-M1_58_:HLA-A*0201 complex. As a result, in the context of our discussion, we can describe IAV-M1_58_:HLA-A*0201 as a spicy complex. The lack of a prominent peptide feature might facilitate the selection of some public TCRs, as indeed observed experimentally (24). But the pMHC-specific pocket allows the selection of a broad TCR repertoire, in the same way as for spicy peptides. Once again, these findings highlight the fact that in most cases we cannot discuss T-cell activation or T-cell cross-reactivity only in terms of peptide-targets, since the key features for recognition might come from the unique combined structure of the pMHC complex.

### 1.5 Local Structural Differences among pMHC Complexes Can Account for Limited Cross-reactivity and Lack of Reciprocity

**Figure 4:**
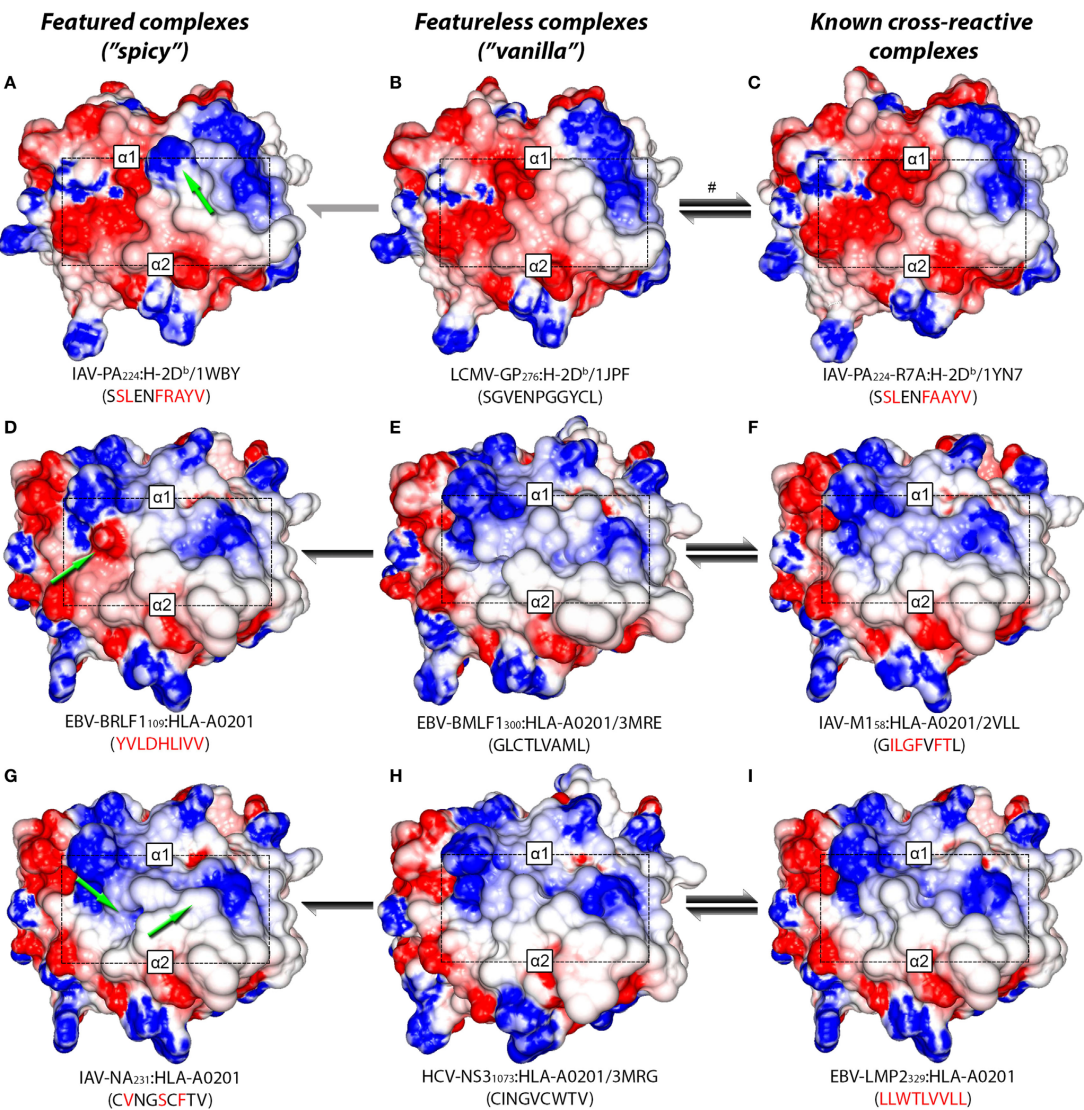
Structural similarity between cross-reactive complexes. TCR-interacting surfaces of selected pMHC complexes were computed with Grasp2 (64). The rows correspond to different datasets of cross-reactive complexes. The central column indicates the reference (cognate) complex in each row (**B,E,H**). The left column indicates a known complex with limited cross-reactivity (**A,D,G**), while the right column indicates a highly cross-reactive complex (**C,F,I**). MHC heavy chain domains *α*1 and *α*2 are indicated in each complex, as well as the region corresponding to the peptide (black rectangle). Colors indicate the range of the electrostatic potential over the surface, from −5 kT/e (red) to +5 kT/e (blue). Complex information and peptide sequence are depicted below each pMHC. For crystal structures, the corresponding PDB ID is also provided. Complexes with no published crystal structure were modeled (see Methods and Resources). Peptide sequences in each line indicate mutations in relation to the corresponding reference peptide (central column). Green arrows highlight “spicy” features of peptides with “limited” cross-reactivity (left column). Black and gray arrows indicate the intensity and preferred directionality of cross-reactivities observed *in vitro*. The symbol # indicates a cross-reactivity that is suggested by our structural analyses, but that was not yet tested experimentally.

In a recent study, Wlodarczyk et al. (17) described a weak cross-reactivity between IAV-PA_224_:H-2D*^b^* and a heterologous complex displaying a peptide derived from lymphocytic choriomeningitis virus (LCMV-GP_276_:H-2D*^b^*, see Table S1 in Supplementary Material). Since crystal structures are available for both complexes, we can visually compare their TCR-interacting surfaces (Figures 4A–C). Notably, LCMV-GP_276_:H-2D*^b^* differs from IAV-PA_224_:H-2D*^b^* by not having the featured arginine at P7. As expected, using our structure-based hierarchical clustering, we can see greater proximity (i.e., structural similarity) between LCMV-GP_276_:H-2D*^b^* and IAV-PA_224_-R7A:H-2D*^b^*, than between these complexes and the wild-type (IAV-PA_224_:H-2D*^b^*) or the non-cross-reactive complex IAV-NP_366_:H-2D*^b^* (Figure S5 in Supplementary Material). As described by Wlodarczyk et al. (17), cross-reactivity between GP_276_:H-2D*^b^* and IAV-PA_244_:H-2D*^b^* was weak and showed a preferential directionality. From the pool of T-cells recognizing GP_276_:H-2D*^b^* (primer) it was possible to extract T-cells that also recognize IAV-PA_224_:H-2D*^b^* (i.e., heterologous challenge). However, the reverse experiment was not successful.

Taken together, these results allow us to postulate that immunization with IAV-PA_224_ stimulates a pool of T-cells dominated by clones with high specificity to the spicy feature (in this case, a peptide feature: the R at P7). By challenging with a heterologous peptide that lacks this prominent feature, we would most likely fail to find a T-cell clone that can also recognize the heterologous vanilla peptide-target (e.g., LCMV-GP_276_). However, by using the vanilla peptide as a primer, we would start from a population of T-cells that is less diverse (i.e., dominated by public TCRs) but more cross-reactive. These TCRs are primarily engaging with (self) MHC structural features; some of these clones might also recognize the heterologous spicy peptide (IAV-PA_224_), regardless of the prominent amino acid residue at P7. We believe this recognition might involve some adjustment of the CDR loops around the center of the peptide, as recently discussed by Adams et al. (29). Naturally, some TCRs will not be able to undergo such adjustment and will not show cross-reactivity. We also hypothesize that the “stronger” the spicy feature (or the combination of diverging features), the stronger the directionality and the lower the likelihood of cross-reactivity. Conversely, we believe stronger cross-reactivity should be observed between very similar pMHC complexes, regardless of directionality. For instance, stronger cross-reactivity should be observed between LCMV-GP_276_:H-2D*^b^* and the mutated IAV-PA_224_-R7A:H-2D*^b^*, than with the wild-type (Figure 1C).

Additional examples supporting this theory can also be found in the context of human MHCs. By the time of our analysis, out of the 9 virus-derived peptides included in our HLA-A*02:01-restricted network (Figure 1B), only 4 had available crystal structures. We modeled the remaining complexes and performed a hierarchical clustering (Figure S6 in Supplementary Material). As expected, the cross-reactive peptide-targets EBV-BMLF1_300_, IAV-M1_58_, HCV-NS3_1073_, HIV-GAG_77_, and EBV-LMP2_329_ were clustered together (see edge 5 in Figure S6 in Supplementary Material). These last two structures were actually the most similar pair of structures inside this cluster, in agreement with previous clustering results from our group (46).

**Figure 5:**
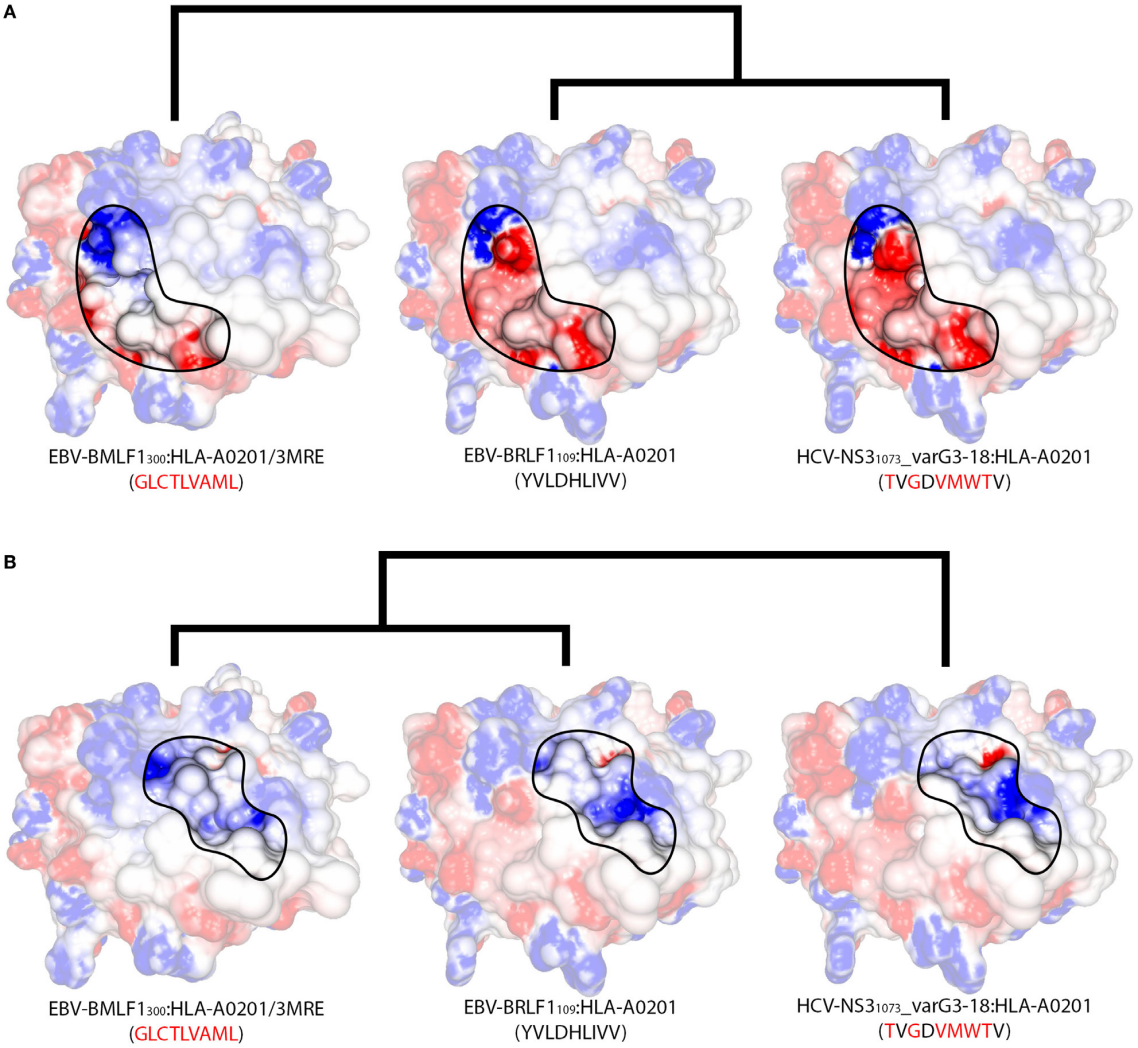
Structural features of different regions produce alternative clusters. Schematic representation of the structural relationships among three HLA-A*02:01-restricted complexes displaying the virus-derived peptides EBV-BMLF1_300_, EBV-BRLF1_109_, and HCV-NS3_1073__varG3-18. Peptide sequences indicate the differences in relation to EBV-BRLF1_109_. **(A)** Focusing the analysis on the region in contact primarily with the TCR’s V*α* domain (as in Figure S1E in Supplementary Material), we observe greater structural similarity between EBV-BRLF1_109_ and HCV-NS3_1073__varG3-18, while EBV-BMLF1_300_ stands out as an unrelated complex. Cross-reactivity between EBV-BRLF1_109_ and HCV-NS3_1073__varG3-18 is suggested by our structural analyses but has not yet been tested experimentally. **(B)** Focusing the analysis on the region in contact primarily with the TCR’s V*β* domain (as in Figure S1F in Supplementary Material), the three complexes become much more similar, with slightly bigger topographical differences for HCV-NS3_1073__varG3-18. Cross-reactivity from EBV-BMLF1_300_ to EBV-BRLF1_109_ has been observed experimentally, in this preferred direction. Although both TCR domains are interacting with the pMHC surface at the same time, there is experimental evidence that one of the domains can be more critical than the other to recognize a given complex (24). The highlighted areas on the pMHC surfaces were arbitrarily defined, for illustration purposes, and do not correspond to the footprint of any particular TCR. In the same way, the heights of the edges in the dendrogram do not capture the actual “distances” among the complexes. The corresponding PDB code is provided for crystal structures; remaining complexes were modeled (see Methods and Resources). The colors over the surfaces indicate the range of charge distribution, from −5 kT/e (red) to +5 kT/e (blue). Additional information on the displayed peptides can be found in Table S1 in Supplementary Material.

Two non-cross-reactive variants of HCV-NS3_1073_ derived from HCV genotype 3, previously referred to as G3-14 and G3-18 (46, 60), fell in separate branches. Despite being the outermost branch of the main cluster (see edge 6 in Figure S6 in Supplementary Material), the small distance between G3-14 and the cross-reactive targets suggest that cross-reactivity with this HCV-derived escape variant might be observed depending on the T-cell population tested. Interestingly, the complex presenting EBV-BRLF1_109_ falls in the same branch as G3-18, which is far from its cross-reactive target (EBV-BMLF1_300_). This HCA result was due to a negatively charged spot in the surface of the EBV-BRLF1_109_:HLA-A*0201 complex, which was not seen in its cross-reactive counterparts (Figures 4D–F). If we remove from our analysis this negatively charged spot, EBV-BRLF1_109_ is clustered with EBV-BMLF1_300_ (Figure 5). Note that we have had access to a yet unpublished crystal structure of EBV-BRLF1_109_:HLA-A*0201, recently resolved by the team of Dr. Lawrence Stern (UMass Medical School, MA, USA), which confirms the existence of the outstanding negatively charged spot observed in our model (Song I, personal communication, June 2017).

Similar to the situation described for IAV-PA_224_, cross-reactivities involving EBV-BRLF1_109_ feature several peculiarities. For instance, they are not observed for most T-cell populations and normally respect a given directionality, from EBV-BMLF1_300_ to EBV-BRLF1_109_ (22). EBV-BRLF1_109_-specific T-cells recovered from EBV-immune individuals and expanded *in vitro* in the presence of the cognate peptide present higher affinity/avidity in TCR/pMHC interaction. Note that these cells are not cross-reactive with EBV-BMLF1_300_. On the other hand, EBV-BMLF1_300_-specific T-cells expanded *in vitro* in the presence of the cognate peptide might also recognize EBV-BRLF1_109_ (22). Further expansion of this population with the heterologous peptide (i.e., EBV-BRLF1_109_) produces (cross-reactive) EBV-BRLF1_109_-specific T-cells with lower affinity/avidity in TCR/pMHC interaction (data not shown).

It is known that TCRs usually interact with pMHCs using a “canonical” binding mode (25, 51, 65, 66), but it was shown that a given TCR can preferentially use distinct amino acid residues to come in contact with different complexes (67) or even modify its CDR loops to accommodate different peptides (68). Therefore, it can be argued that immunization with a spicy peptide (such as IAV-PA_224_ or EBV-BRLF1_109_) will trigger a highly polyclonal T-cell response, with a broad spectrum of TCR specificities. Some of these are less specific to the homologous target, and more cross-reactive with other peptides, probably by establishing an interaction “focused” on surface regions that are shared among these targets (Figure 4). On the other hand, some of these cells present higher affinity/avidity with this homologous peptide, by establishing an interaction “focused” on unique features of its surface (Figure 5). In turn, cross-reactivity between a spicy and a vanilla peptide depends on which T-cell populations are being tested.

We here hypothesize that, despite different TCRs can share a similar TCR footprint or even interact with the same pMHC amino acid residues, each TCR has a specific “interaction profile.” That is, some TCR/pMHC interactions are more important than others for triggering the T-cell response, and this interaction profile is specific to each TCR (Figures S1E,F in Supplementary Material). Knowing the specific hot-spots of a cognate pMHC, i.e., the aforementioned “focus” of the TCR, would be key to predict cross-reactivity against heterologous pMHC targets. Moreover, although we tend to think of these hot-spots as pMHC amino acid residues, we need to expand this concept to account for more subtle features of the TCR/pMHC interaction (e.g., pockets, hydrogen bonds, van der Waals contacts, and coordination of water molecules) (24).

### 1.6 T-Cell Cross-reactivity Can Be Triggered by High-Affinity Interactions with Specific Structural Features of the pMHC Complex

We previously suggested that T-cells expanded in response to a vanilla peptide should be intrinsically more cross-reactive, since they are focused on patterns shared across different pMHC complexes. Conversely, T-cells expanded in response to a spicy peptide are expected to be less cross-reactive in general, since most heterologous peptides would lack the spicy feature that is the focus of the response. However, these cells should still be cross-reactive with peptides having the spicy feature, in some cases regardless of other evident differences.

In fact, studies in cancer immunotherapy show that mutations leading to increased affinity of a given TCR-peptide interaction can actually increase cross-reactivity (38–40, 69). We hypothesize that although not changing the overall TCR footprint, such mutations can change the interaction profile of the TCR. In other words, the enhanced peptide-specific interaction becomes much more important for T-cell activation than the additional pMHC interactions, and any heterologous pMHC sharing the structural feature recognized by this enhanced TCR can become a cross-reactive target.

Further evidence for this hypothesis comes from a recent publication by Adams et al. (29). Using a carefully designed experimental approach, the authors investigated cross-reactive peptides showing limited sequence identity with the reference cognate peptide (restricted to H-2K*^d^*). Despite apparent sequence diversity among peptides recognized by the probe TCR, closer analysis revealed a repeated focus on structurally and chemically similar elements of the peptides. For instance, the authors describe a preferred interaction with hydrophobic amino acid residues at P7; particularly phenylalanine. The authors refer to this amino acid residue as a peptide hot-spot for cross-reactivity, which in combination with some germline-mediated interactions greatly constrains the actual pool of potential cross-reactive pMHC targets (for the probe TCR). They also relate this description of the TCR/pMHC interaction with a more general feature of protein-protein interactions: a few energetically important contacts (usually in the center), surrounded by weaker and more diverse peripheral interactions. In the context of our discussion, we could see the phenylalanine at P7 as a spicy feature of the cognate peptide and the most important contact in the interaction profile of the probe TCR.

As mentioned earlier, we have previously described cross-reactivity between peptides with no sequence similarity, but with remarkably similar TCR-interacting surfaces (Figures 4G–I). The results described by Song et al. (24) provide an interesting example in which even greater variability can be anticipated. If the main feature for TCR recognition is a pocket defined by the peptide in the MHC cleft (e.g., IAV-M1_58_:HLA-A*0201), we can expect that such “pocket-specific” T-cells will be cross-reactive to other pMHC complexes having a similar pocket, maybe regardless of other differences in the TCR-interacting surface. For instance, it is possible for a completely unrelated pMHC (with a different peptide sequence and/or MHC allotype) to have a very similar pocket and, therefore, be a cross-reactive target for IAV-M1_58_-specific T-cells.

As also discussed by Adams et al. (29), the implications of such “hot-spots” for cross-reactivity prediction are clear. A superficial look at the sequence diversity of cross-reactive peptides might suggest a completely promiscuous recognition, even considering a single TCR. The picture becomes even more complex if on top of that we start considering different pools of T-cells or the *in vivo* response of different individuals, which adds variability given to private specificity and immunological history. This complex picture helps understand the challenge of comparing results from different studies and drawing general conclusions about T-cell cross-reactivity. On the other hand, the characterization of cross-reactivity hot-spots and TCR-specific interaction profiles should allow us to focus our research and make progress for meaningful cross-reactivity predictions.

In fact, Arber et al. (56) published a study that goes in this very direction. They combined T-cell assays and computational analysis to evaluate T-cell cross-reactivity of different clones in the context of cancer immunotherapy. Based on IFN-*γ* production against a panel of alanine-exchanged variants of the cognate peptide, they defined T-cell-specific sequence motifs. These motifs were meant to capture T-cell-specific cross-reactivity hot-spots; they were later used for a sequence-based screening of potential cross-reactive targets in the human proteome. A number of positive hits were selected and tested experimentally, confirming that one T-cell line was much safer (i.e., less cross-reactive) than the other. The scope of this screening was still limited, not accounting for structural information of the pMHC or other potentially relevant features (14, 24, 70). Nevertheless, it provides us with an example of the type of framework that would be required for T-cell-specific prediction of potential cross-reactive targets.

### 1.7 Conclusions and Implications for Cancer Immunotherapy

Several immunotherapy trials are currently underway in a number of different tumor types to target tumor-associated peptides (71), including the melanoma-associated antigens MAGE-A3 and MART-1. These tumor antigens are expressed by multiple tumor types (39) but are not expressed by most normal tissues. Since MART-1 is highly expressed in both melanoma and normal melanocytes, MART-1 TCR-based therapies have led to antitumor responses concurrent with vitiligo and melanocyte destruction in the eye and inner ear, side effects that could be relieved with steroid administration (72). However, more severe safety issues with other TCR-based therapies have raised major concerns about this approach (33, 73, 74). As mentioned earlier, fatal adverse events were reported following adoptive transfer of TCR-transduced T-cells targeting complexes displaying the MAGE-A3 peptide (39–42). In two of these patients, unexpectedly severe cardiac toxicity was attributed to recognition of a completely unrelated peptide. This heterologous peptide-target was derived from the self protein Titin and displayed by HLA-A*01:01 at the surface of healthy cardiac cells (43). As discussed by Stone et al. (38), T-cell cross-reactivity becomes specially relevant in the context of affinity-enhanced TCRs. Approaches like this are becoming more popular through the use of chimeric antigen receptors (CARs) (71). However, as reported by van den Berg et al. (41), severe off-target reactions can occur even without TCR-affinity enhancement. And this adds a layer of concern on top of toxicity and autoimmunity that might occur even with the use of autologous tumor infiltrating T-cells (72, 75). Moreover, as highlighted in our review, T-cell cross-reactivity seems to be rather the rule than the exception. Therefore, despite all mechanisms of central and peripheral tolerance (76), off-target toxicity mediated by T-cell cross-reactivity must be a concern in any TCR-based immunotherapy. However, the risk for off-target toxicity will differ depending on which specific form of therapy is being used.

Our study corroborates the idea that structural similarity among pMHC complexes is one of the main features driving the likelihood of cross-reactive T-cell responses. Cross-reactivity is very likely to be observed between two structurally identical complexes, for most T-cell lines recognizing one of the complexes, and in both directions. On the other hand, finding a T-cell line capable of recognizing two completely different pMHC complexes is highly unlikely. However, in most cases, two complexes will have common features but also different ones. In this situation, cross-reactivity can only be assessed by the level of pMHC structural similarity, as an intrinsic likelihood. However, its occurrence, intensity and directionality will be driven by the specific T-cell population stimulated by the first target and selectively expanded after heterologous challenges.

In the context of polyclonal T-cell populations, this outcome is mostly a consequence of private specificity and immunological history (19, 20, 32). Therefore, predicting patient cross-reactivity in response to immunization, infection or tissue transplantation is very challenging. Even knowing the peptide-targets and the MHC alleles of the patient, and having the perfect tools to estimate intrinsic cross-reactivity probabilities, we would still lack information on the available T-cell repertoire and the interaction profile of the dominating T-cell line. On the other hand, some problems in cancer immunotherapy offer a much more constrained scenario. In the context of TCR-based immunotherapies, researchers know which TCR is being used to recognize the tumor-derived peptide-target and can ensure that this will be the dominating population during treatment. By narrowing our analysis to a particular therapeutic T-cell line, we can limit the scope of cross-reactivity to structural features of the targeted pMHC; more specifically, to hot-spots that are the focus of the therapeutic TCR.

Therefore, we advocate that an important goal of structural analyses in the field of immunotherapy should be the characterization of the TCR-specific recognition profile. This profile should be a refinement of a more general TCR footprint, highlighting which pMHC structural features are more important for triggering this particular T-cell. In turn, this information can be used to guide large-scale *in silico* screenings, based on a combination of structural and sequential information. Currently, no tool can perform such screenings in a personalized fashion, especially when considering the diversity of MHC alleles in the human population (5). However, T-cell cross-reactivity prediction will soon be enabled by advances in both pMHC structural modeling and TCR sequence analyses.

On the pMHC side, the combination of new modeling methods (57, 77) and structural clustering approaches (48, 78, 79) will allow considering structural information for larger datasets, regardless of whether experimental data are available. On the TCR side, recent reports have shown exciting results in the identification of conserved CDR motifs that can be directly linked to TCR specificity (11, 80). In time, we should be able to define T-cell-specific interaction profiles based on the sequence of the CDR regions of the TCR of interest.

Finally, better understanding of all subtle structural features relevant to TCR/pMHC engagement (11, 24) and their contributions to TCR binding affinity (37, 81–83) will also facilitate efforts toward TCR engineering and rational design (37, 84–86). The TCR-specific interaction profile can inform computer-aided efforts to increase TCR affinity to tumor-specific peptides, while reducing the risk for off-target toxicity. Hopefully, the combined use of these new technologies will soon allow researchers to predict and validate potentially dangerous cross-reactivities in the early stages of therapy development, guiding additional procedures to achieve safer TCR-based immunotherapies. Despite the overall complexity of the subject, urgent needs in cancer immunotherapy are pushing the discussion forward and should pave the way for many additional contributions to other areas of human health.

## 2 Methods and Resources

### 2.1 Experimental Data on Cross-reactivity Networks

Cross-reactivity networks depicted in Figure 1 were compiled from previously published experiments. Most data were made available by Cornberg et al. (22), who first presented these networks. The authors also described an escape variant of LCMV-NP_205_ with a V3A substitution (21), suggested its sequence similarity with peptides from old world arenaviruses (MOPV-NP_205_ and LASV-LNP_209_) and finally solved its 3D crystal structure in the context of H-2K*^b^* (62). This study with murine cross-reactivities was further explored by Shen et al. (28). The murine H-2D*^b^*-restricted network was depicted with data from Wlodarczyk et al. (17).

Cornberg et al. (22) also described a human HLA-A*02:01-restricted network. We expanded this network by including a cross-reactive target prospected through structural *in silico* analysis (46) and already confirmed experimentally (47), as well as two non-cross-reactive targets described by Fytili et al. (60). These tested non-cross-reactive targets were included both in human and murine cross-reactivity networks to provide further experimental information to guide our structure-based analysis.

A careful verification of peptides’ information was performed to determine the correct protein name and peptide position, providing an updated reference for future studies (Table S1 in Supplementary Material). Curated information from Uniprot (87) was used as the main reference, and GenBank (88) was also consulted. References to the Immune Epitope Database (IEDB) (89), the Protein Data Bank (PDB) (90), and the CrossTope Database (91) were also provided, when available.

### 2.2 Crystal Structures

Crystal structures were obtained from the Protein Data Bank (PDB) (90) and revised as needed using the PyMOL Viewer (92). The resulting pMHC structure was submitted to a short energy minimization with the Gromacs 4.5.1 package (93).

Note that 3TID is referred to as the crystal structure of LCMV-GP_34_:H-2K*^b^* complex, despite presenting an amino acid exchange at P8 (LCMV-GP_34_-C8M). According to the authors who described the structure (28), this exchange has no significant impact on TCR/pMHC interactions and this C8M variant was used in previous studies as an “equivalent” to the wild-type sequence. Here, sequence divergence between LCMV-GP_34_ and LCMV-GP_34_-C8M is indicated in Figure 3, but 3TID was considered as the crystal structure of LCMV-GP_34_ for all structure-based analyses.

### 2.3 Modeled Structures

Peptide–MHC complexes without published crystal structures were predicted using the DockTope webserver (57). Briefly, a reference crystal structure of the MHC allotype of interest (without its ligand) was used as a receptor (“MHC_donor”) for a molecular docking with Autodock Vina 1.1.2 (94). The input ligand structure was produced by mutating a peptide structure obtained in the context of the same MHC allotype (“Peptide_pattern”). The resulting pMHC structure was then refined through a full atom energy minimization step with the Gromacs 4.5.1 package (93). A new docking search was performed with only the peptide side chains being flexible. This automated approach for pMHC structure prediction was largely validated against available crystal structures (57).

### 2.4 Electrostatic Potential Calculation and Image Analysis

Electrostatic potential over the TCR-interacting surface of pMHCs (for both crystals and models) was calculated using Delphi (95), through the molecular viewer software GRASP2 (64). Automated scripts were used to prepare the structures for this analysis, allowing all pMHCs to be observed in the same fixed orientation. Images of the TCR-interacting surfaces were saved and imported to the ImageJ 1.46r software (National Institute of Health, USA, https://imagej.nih.gov/ij/). Using preexisting classes from ImageJ, our team adapted a plugin to import RGB values from predetermined regions over the pMHC surface (as in Figure S1D in Supplementary Material), following a previously described protocol (46, 47). Values were exported as “csv” tables and used as input for hierarchical cluster analysis.

### 2.5 Hierarchical Cluster Analysis

In this study, hierarchical clustering was used as a tool to assess structure-based similarity among pMHC complexes. Input values were extracted from the images of the TCR-interacting surfaces (see section 2.4). Hierarchical clustering was performed with pvclust (61), an R package for assessing the uncertainty in hierarchical clustering. The “average” linkage method was used with “correlation” distance, and the number of bootstrap replications was set to 10,000. Results were plotted as dendrograms with approximately unbiased (AU) and bootstrap probabilities (BP) p-values. BP values are calculated by normal bootstrap resampling, and AU values are computed through multiscale bootstrap resampling, which is considered a better approximation to unbiased p-value (61). SEs for AU p-values were obtained with seplot, presenting values lower than 0.01 for all clusterings performed.

## Author Contributions

DA, GV, MC, and LS suggested the initial idea behind this work. DA, MR, MS, and GV conceived the experiments. DA selected the dataset and MS curated the information on selected peptides. DA, MR, and MM conducted the modeling and clustering experiments. MF adapted the ImageJ plugin and helped with the extraction of the values for clustering. LK revised clustering experiments and algorithmic choices. DA, MR, GV, MS, MC, and LS analyzed and interpreted the results. GL contributed with the applications to immunotherapy and the review of related literature. DA wrote the manuscript. All the authors reviewed and approved the final manuscript.

## Conflict of Interest Statement

The authors declare that the research was conducted in the absence of any commercial or financial relationships that could be construed as a potential conflict of interest.

## Supplementary Material

Table S1 List of all studied complexes. Identification and source information for each peptide and MHC, as well as corresponding access codes to relevant databases.

Figure S1 The TCR-interacting surface and the proposed TCR interaction profiles. **(A)** Top view of a pMHC complex depicting the MHC-receptor as *cartoon* (gray) and the peptide–ligand as *sticks* (pink). **(B)** Top view of the same pMHC complex, depicting the exposed surface of the MHC (gray) and the exposed surface of the peptide (pink). **(C)** The combined surface of the pMHC complex, with the colors indicating the range of charge distribution over the surface from −5 kT/e (red) to +5 kT/e (blue). This is the “face” of the pMHC exposed for TCR recognition, referred to as the *TCR-interacting surface*. **(D)** The TCR binds to the pMHC in a conserved orientation: the TCR’s variant domain V*α* primarily interacts with the N-terminal portion of the peptide, while the V*β* domain primarily interacts with the C-terminal portion of the peptide. This area of TCR/pMHC interaction, in a particular docking angle, is referred to as the *TCR footprint*. **(E)** Schematic representation of a *TCR-specific interaction profile* over the pMHC surface. Colored boxes indicate “hot-spots” for cross-reactivity (green) and secondary contacts that also contribute to TCR binding affinity (yellow). **(F)** Schematic representation of a different interaction profile, displayed by a different TCR that still shares the same general TCR footprint. Both depicted profiles are simplified schematic representations and do not represent known interactions of a any particular TCR.

Figure S2 Crystal-based H-2K*^b^*-restricted clustering. Structure-based hierarchical clustering performed with pvclust (61). Each putative cluster is represented by a specific edge (gray numbers), in order of increasing heights (y axis). Cluster confidence is measured with two p-values, approximately unbiased (AU) and bootstrap probabilities (BP). Lines highlighted in purple indicate structures with greater structural similarity (as represented in Figure 1). Peptide abbreviation and corresponding PDB code for each crystal structure (in blue) are provided. *Crystal structure 3TID was used to represent LCMV-GP_34_, despite presenting a C8M exchange (see Methods and Resources).

Figure S3 TCR-interacting surfaces of predicted cross-reactive targets. Regions with positive (blue) and negative (red) charges are represented with a scale from −5 to +5 kT/e. Information on the corresponding peptide and MHC restriction is provided below each complex. Amino acid exchanges in relation to LCMV-GP_118_ are indicated. Great structural similarity is observed between these two complexes, both in terms of topography and electrostatic potential over the TCR-interacting surface. Note that other subtle structural differences might exist but are not well captured by this representation of the complexes.

Figure S4 Specific interaction with a prominent peptide amino acid. **(A)** Surface of the IAV-PA_224_:H-2D*^b^* complex (spicy peptide) according to a crystal structure obtained in the absence of the TCR (PDB code 1WBY). **(B)** Surface of the same complex according to a crystal structure obtained in the presence of the TCR (PDB code 3PQY). **(C)** Cartoon depiction of the 3PQY structure highlighting TCR amino acid residues that interact directly with a prominent arginine at the peptide (R7), forming a negatively charged cavity. Side chain of amino acid R7 is depicted in *ball and stick*. TCR and MHC domains are indicated, and green arrows highlight the location of amino acid residue R7. Electrostatic potentials were computed with Grasp2 (64).

Figure S5 Crystal-based H-2D*^b^*-restricted clustering. Structure-based hierarchical clustering performed with pvclust. Each putative cluster is represented by a specific edge (gray numbers), in order of increasing heights (y axis). Cluster confidence is measured with two p-values, approximately unbiased (AU) and bootstrap probabilities (BP). Peptide abbreviation and the respective PDB code for each crystal structure (in blue) are provided. Lines highlighted in purple indicate structures with greater structural similarity (as represented in Figure 1).

Figure S6 Extended HLA-A*02:01-restricted clustering. Structure-based hierarchical clustering performed with pvclust. Each putative cluster is represented by a specific edge (gray numbers), in order of increasing heights (y axis). Cluster confidence is measured with two p-values, approximately unbiased (AU) and bootstrap probabilities (BP). Abbreviation of crystal structures includes their PDB code (in blue), while “Mod” indicates modeled structures. Lines highlighted in purple indicate structures with greater structural similarity (as represented in Figure 1).
