# Supplementary figures and images for: Interpreting T-Cell Cross-reactivity through Structure: Implications for TCR-Based Cancer Immunotherapy
Source: Front Immunol. 2017 Oct 4;8:1210. doi: 10.3389/fimmu.2017.01210 (PMC5632759; doi:10.3389/fimmu.2017.01210)

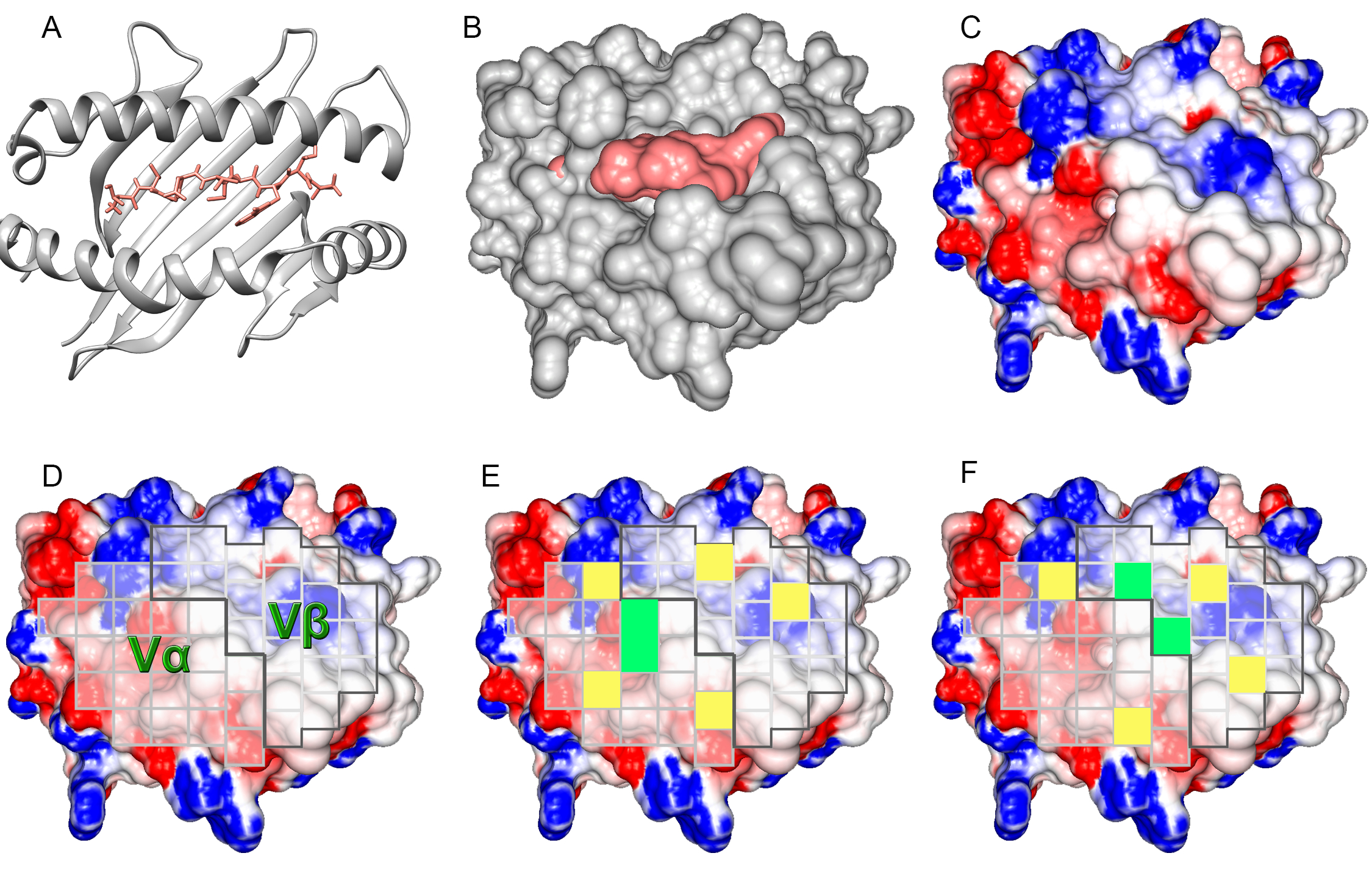

Supplement: Figure S1 — The TCR-interacting surface and the proposed TCR interaction profiles. (A) Top view of a pMHC complex depicting the MHC-receptor as cartoon (gray) and the peptide–ligand as sticks (pink). (B) Top view of the same pMHC complex, depicting the exposed surface of the MHC (gray) and the exposed surface of the peptide (pink). (C) The combined surface of the pMHC complex, with the colors indicating the range of charge distribution over the surface from −5 kT/e (red) to +5 kT/e (blue). This is the “face” of the pMHC exposed for TCR recognition, referred to as the TCR-interacting surface. (D) The TCR binds to the pMHC in a conserved orientation: the TCR’s variant domain Vα primarily interacts with the N-terminal portion of the peptide, while the Vβ domain primarily interacts with the C-terminal portion of the peptide. This area of TCR/pMHC interaction, in a particular docking angle, is referred to as the TCR footprint. (E) Schematic representation of a TCR-specific interaction profile over the pMHC surface. Colored boxes indicate “hot-spots” for cross-reactivity (green) and secondary contacts that also contribute to TCR binding affinity (yellow). (F) Schematic representation of a different interaction profile, displayed by a different TCR that still shares the same general TCR footprint. Both depicted profiles are simplified schematic representations and do not represent known interactions of a any particular TCR. [file image_1.jpeg]

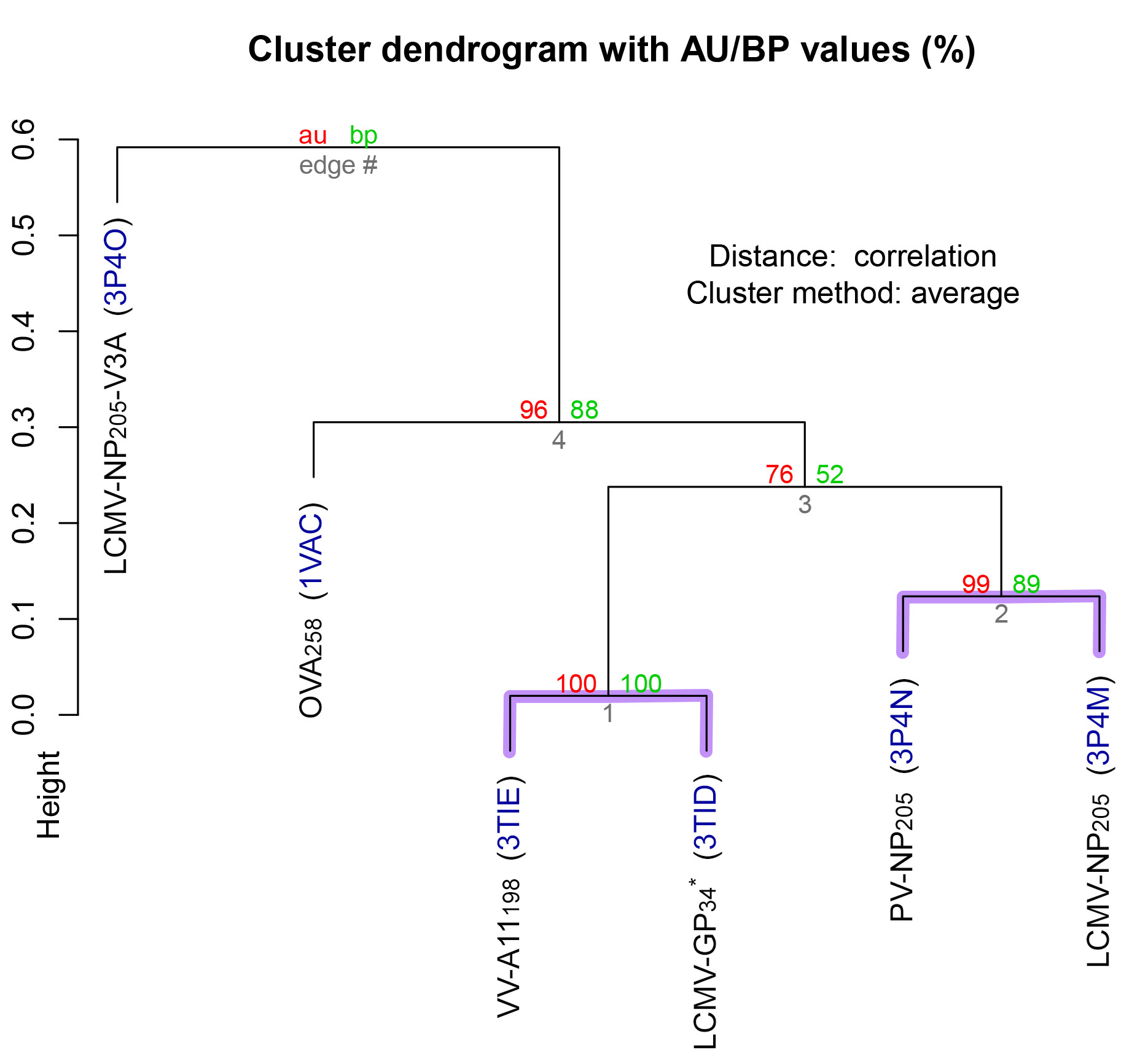

Supplement: Figure S2 — Crystal-based H-2Kb-restricted clustering. Structure-based hierarchical clustering performed with pvclust (61). Each putative cluster is represented by a specific edge (gray numbers), in order of increasing heights (y axis). Cluster confidence is measured with two p-values, approximately unbiased (AU) and bootstrap probabilities (BP). Lines highlighted in purple indicate structures with greater structural similarity (as represented in Figure 1). Peptide abbreviation and corresponding PDB code for each crystal structure (in blue) are provided. *Crystal structure 3TID was used to represent LCMV-GP34, despite presenting a C8M exchange (see Methods and Resources). [file image_2.jpeg]

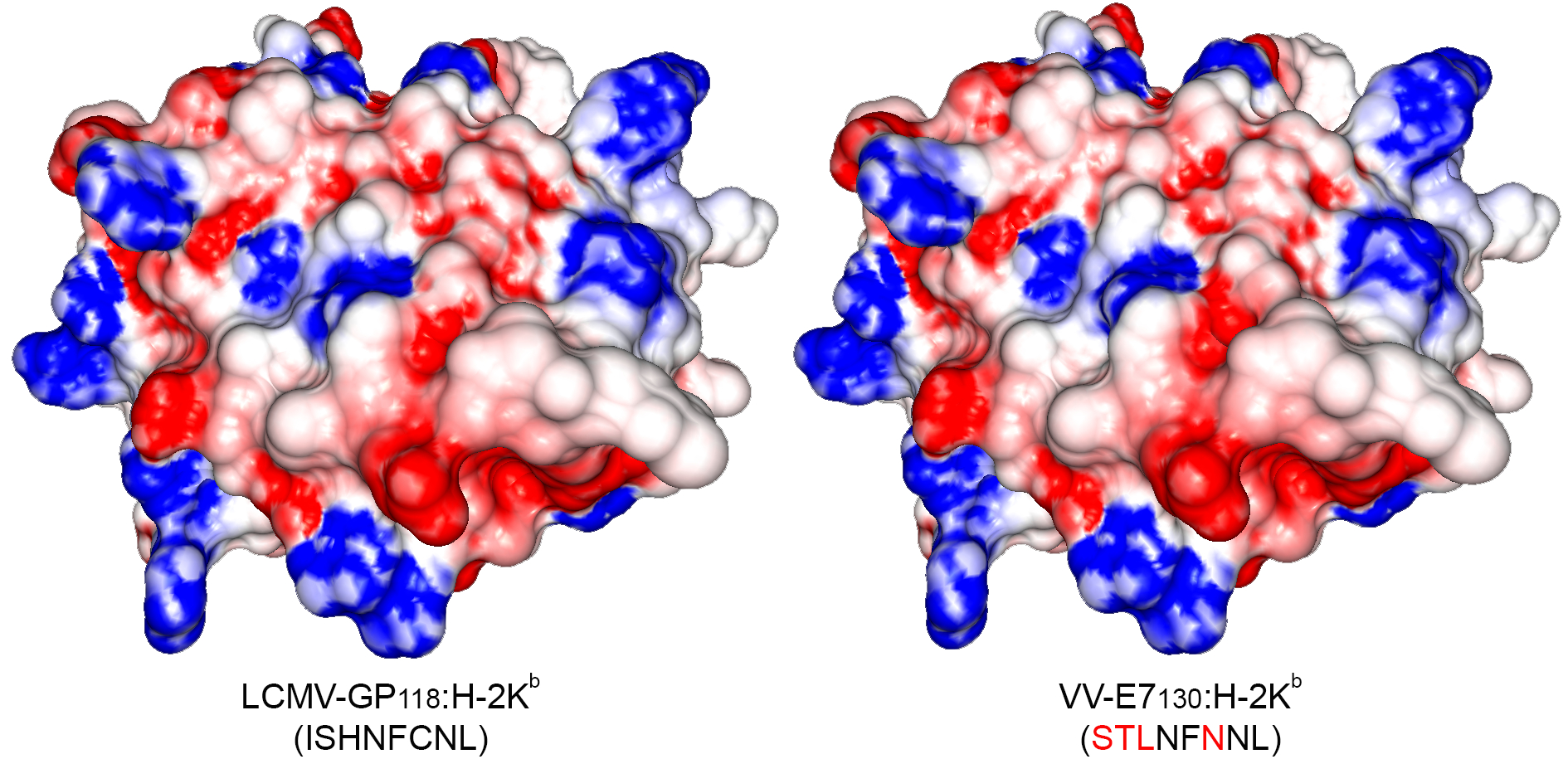

Supplement: Figure S3 — TCR-interacting surfaces of predicted cross-reactive targets. Regions with positive (blue) and negative (red) charges are represented with a scale from −5 to +5 kT/e. Information on the corresponding peptide and MHC restriction is provided below each complex. Amino acid exchanges in relation to LCMV-GP118 are indicated. Great structural similarity is observed between these two complexes, both in terms of topography and electrostatic potential over the TCR-interacting surface. Note that other subtle structural differences might exist but are not well captured by this representation of the complexes. [file image_3.jpeg]

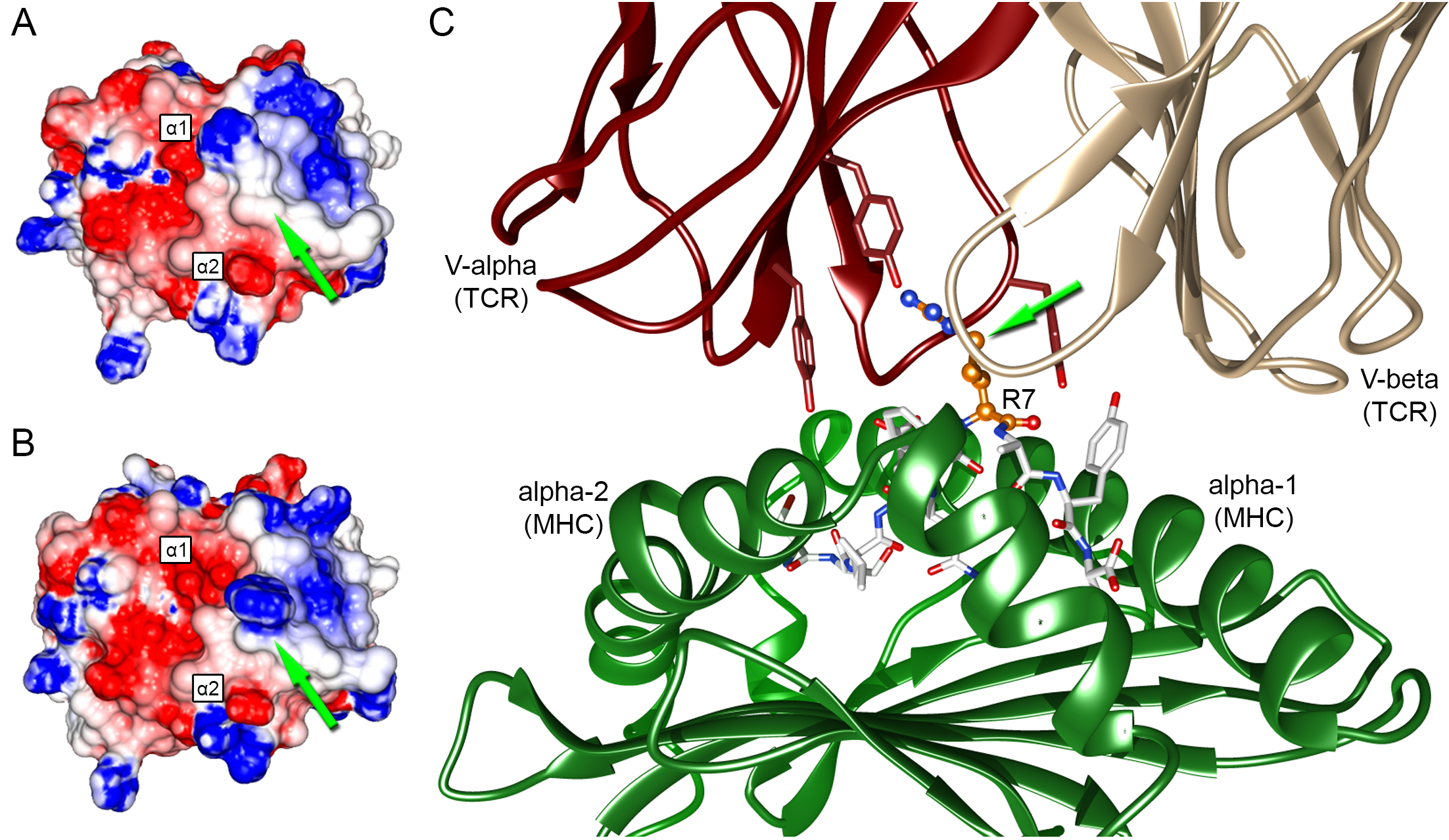

Supplement: Figure S4 — Specific interaction with a prominent peptide amino acid. (A) Surface of the IAV-PA224:H-2Db complex (spicy peptide) according to a crystal structure obtained in the absence of the TCR (PDB code 1WBY). (B) Surface of the same complex according to a crystal structure obtained in the presence of the TCR (PDB code 3PQY). (C) Cartoon depiction of the 3PQY structure highlighting TCR amino acid residues that interact directly with a prominent arginine at the peptide (R7), forming a negatively charged cavity. Side chain of amino acid R7 is depicted in ball and stick. TCR and MHC domains are indicated, and green arrows highlight the location of amino acid residue R7. Electrostatic potentials were computed with Grasp2 (64). [file image_4.png]

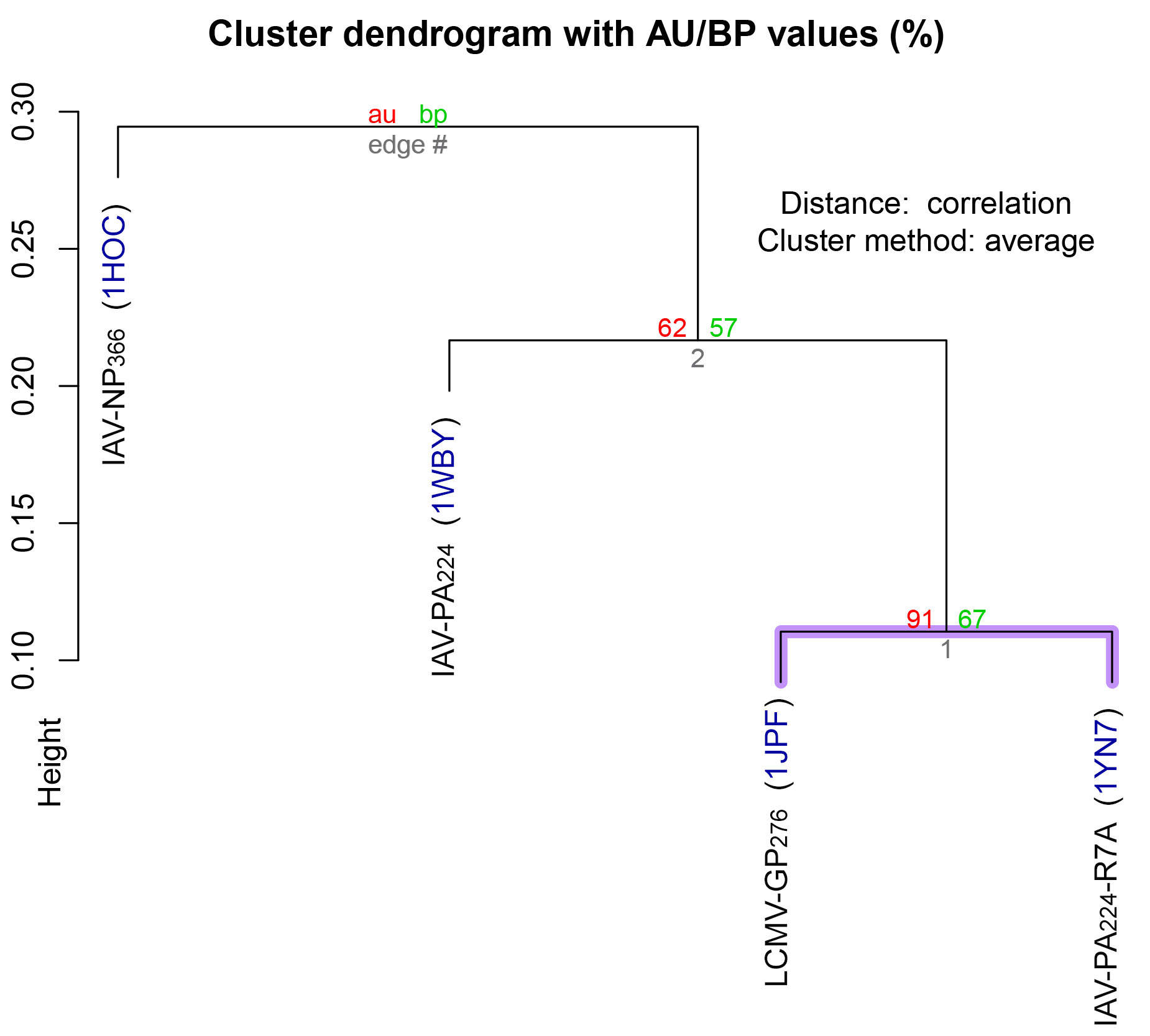

Supplement: Figure S5 — Crystal-based H-2Db-restricted clustering. Structure-based hierarchical clustering performed with pvclust. Each putative cluster is represented by a specific edge (gray numbers), in order of increasing heights (y axis). Cluster confidence is measured with two p-values, approximately unbiased (AU) and bootstrap probabilities (BP). Peptide abbreviation and the respective PDB code for each crystal structure (in blue) are provided. Lines highlighted in purple indicate structures with greater structural similarity (as represented in Figure 1). [file image_5.jpeg]

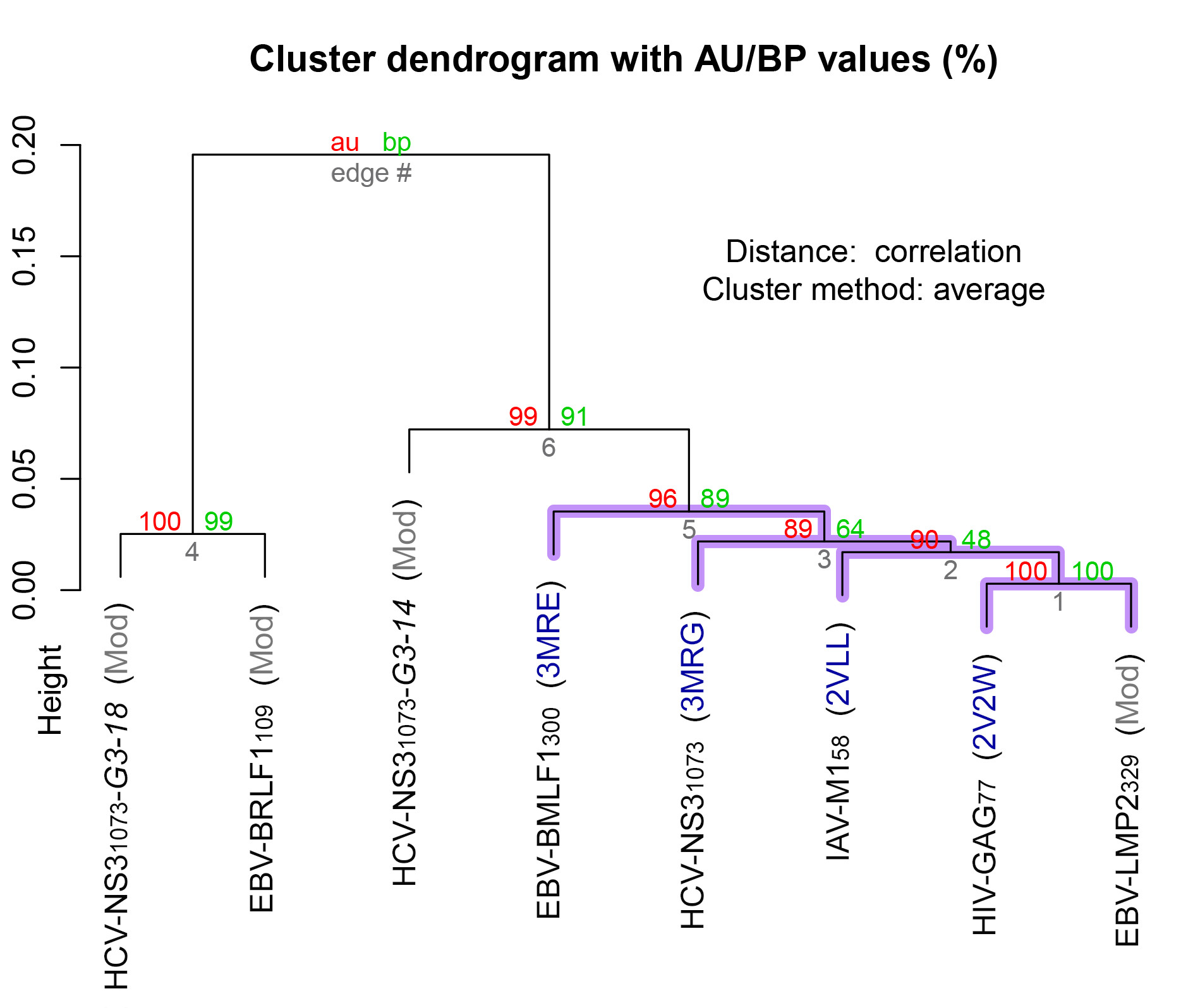

Supplement: Figure S6 — Extended HLA-A*02:01-restricted clustering. Structure-based hierarchical clustering performed with pvclust. Each putative cluster is represented by a specific edge (gray numbers), in order of increasing heights (y axis). Cluster confidence is measured with two p-values, approximately unbiased (AU) and bootstrap probabilities (BP). Abbreviation of crystal structures includes their PDB code (in blue), while “Mod” indicates modeled structures. Lines highlighted in purple indicate structures with greater structural similarity (as represented in Figure 1). [file image_6.jpeg]
